# Human Inner Ear Immune Activity: A Super-Resolution Immunohistochemistry Study

**DOI:** 10.3389/fneur.2019.00728

**Published:** 2019-07-10

**Authors:** Wei Liu, Charlotta Kämpfe Nordström, Niklas Danckwardt-Lillieström, Helge Rask-Andersen

**Affiliations:** Section of Otolaryngology, Department of Surgical Sciences, Uppsala University Hospital, Uppsala, Sweden

**Keywords:** human, inner ear, IBA1, macrophages, structured illumination microscopy

## Abstract

**Background:** Like the brain, the human inner ear was long thought to be devoid of immune activity. Only the endolymphatic sac (ES) was known to be endowed with white blood cells that could process antigens and serve as an immunologic defense organ for the entire inner ear. Unexpectedly, the cochlear and vestibular organs, including the eighth cranial nerve, were recently shown to contain macrophages whose functions and implication in ear disease are somewhat undefined. Here, we review recent inner ear findings in man and extend the analyses to the vestibular nerve using super-resolution structured illumination microscopy (SR-SIM).

**Materials and Methods:** Human ESs and cochleae were collected during surgery to treat patients with vestibular schwannoma and life-threatening petro-clival meningioma compressing the brainstem. The ESs and cochleae were placed in fixative, decalcified, and rapidly frozen and cryostat sectioned. Antibodies against ionized calcium-binding adaptor molecule 1-expressing cells (IBA1 cells), laminin β2 and type IV collagen TUJ1, cytokine fractalkine (CX3CL1), toll-like receptor 4 (TLR4), CD68, CD11b, CD4, CD8, the major histocompatibility complex type II (MHCII), and the microglial marker TEME119 were used.

**Results:** IBA1-positive cells were present in the ESs, the cochlea, central and peripheral axons of the cochlear nerve, and the vestibular nerve trunk. IBA1 cells were found in the cochlear lateral wall, spiral limbus, and spiral ganglion. Notable variants of IBA1 cells adhered to neurons with “synapse-like” specializations and cytoplasmic projections. Slender IBA1 cells occasionally protracted into the basal lamina of the Schwann cells and had intimate contact with surrounding axons.

**Discussion:** The human eighth nerve may be under the control of a well-developed macrophage cell system. A small number of CD4+ and CD8+ cells were found in the ES and occasionally in the cochlea, mostly located in the peripheral region of Rosenthal's canal. A neuro-immunologic axis may exist in the human inner ear that could play a role in the protection of the auditory nerve. The implication of the macrophage system during disease, surgical interventions, and cell-based transplantation should be further explored.

## Introduction

**Figure 1:**
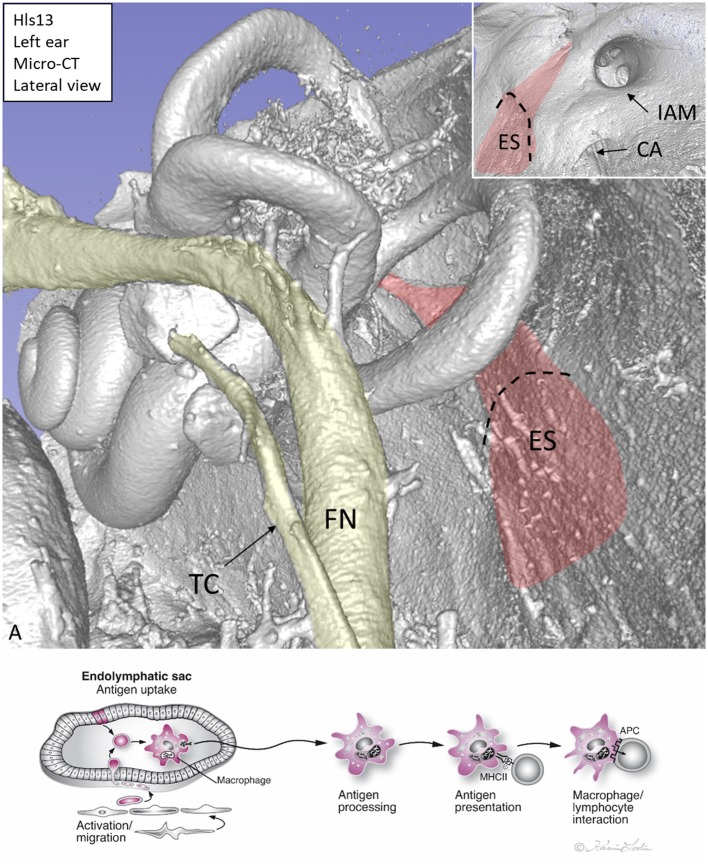
**(A)** Micro-CT, 3D reconstruction of a left human inner ear silicone mold. The ES (red) is located on the posterior slope of the petrous pyramid. It is connected to the rest of the inner ear through the endolymphatic duct. Inset shows the intra-cranial view of the ES. **(B)** Hypothetical representation of scavenger and foreign substance uptake in the human ES. CA, cochlear aqueduct; IAM, internal acoustic meatus; FN, facial nerve; TC, tympanic chorda; MHCII, major histocompatibility complex class type II. APC, antigen-presenting cell.

The human inner ear and its immune activity are difficult to study because it is surrounded by the hardest bone in the body. In fact, the inner ear was long thought to lack immune activity. Immune cells were restricted to the so-called endolymphatic sac (ES), a membranous appendage situated on the posterior slope of the petrous pyramid at some distance from the sensory regions (Figure 1A). The ES contains white blood cells that populate the sub-epithelium and its lumen, and this was exquisitely described by Stacey Guild already in 1927 (1). He managed to maintain the integrity of the ES borders with luminal contents. Various types of leucocytes were observed and later analyses using ultrastructure showed signs of lymphocyte–macrophage interaction and mature plasma cells, suggesting an ongoing immune activity (2, 3). Hypothetically, antigens could reach this area from the respiratory mucosa of the middle ear, cochlear aqueduct, (4) or the vascular system (Figure 1B). A possible entry could be the round window that is enclosed by a thin membrane (<0.1 mm). The human inner ear tissue is extremely vulnerable and needs protection from pervasive infectious intrusions. Experimental results suggest that the ES may collect and neutralize noxious substances but can also exert secondary immune activity (5). Ablation of the ES has been shown to diminish this safeguard and to result in an increased vulnerability (6). Nevertheless, experiments suggested that immune responses are not entirely dependent on the ES. Specific immunity, after antigenic challenge, can be detected in the cochlea even after its ablation, but to a reduced extent (6). This indicates that antigen-presenting cells can also be present in the cochlea (7). Morphologic evidence of immune activity in the human ES was presented by Bui et al. (8). Recently, the immunological capacity of the ES was described through gene arrays (9).

New microscopic techniques have increased our concept of the molecular organization of the human inner ear. Immunohistochemistry was performed using super-resolution structured illumination microscopy (SR-SIM) of well-fixed specimens after mild decalcification (10–12). The ion channel machinery of the lateral wall (“cochlear battery”) was recently analyzed (10, 13). Immune localization of IBA1-positive macrophages was made in the cochlea and ES (12, 14). This verified the existence of a multitude of macrophages in the human inner ear as previously demonstrated by light microscopy and immunostaining of celloidin sections of temporal bones (15). Here, we extended the analysis of the human ES, cochlea, and cochlear and vestibular nerves and ganglia (12, 14). We further analyzed CD4 and CD8 lymphocytes in the cochlea (16) and the ES. This study was a collaboration between neuro-otologists and cell biologists at the University Hospital of Uppsala, Sweden.

## Materials and Methods

### Ethics Statement

Human cochleae were collected during trans-cochlear surgery to remove life-threatening petro-clival meningioma compressing the brainstem. To completely remove the tumors, a petrosectomy was performed that included a postero-inferior re-routing of the facial nerve. Instead of drilling the cochlea away, it was dissected out after approval from the ethical committee and the patient after written consent. The cochlea was immediately fixed according to the techniques described earlier (10, 17, 18). The study of human cochleae was approved by the local ethics committee (Etikprövningsnämnden Uppsala, no. 99398, 22/9 1999, cont. 2003, no. C254/4; no. C45/7 2007, Dnr. 2013/190) and the patients. Written information was given to patients operated for petro-clival meningioma. The patients ranged from 40 to 70 years of age. Their hearing thresholds (pure tone audiometry) were normal, except in a few cases where frequencies showed slightly increased thresholds. At vestibular schwannoma surgery, the ES is routinely drilled away and wasted. The ethical committee approved that such tissue could be collected and directly analyzed histologically without storing personal data.

### Preparation of Human Tissue

Studies of the human cochlea are particularly challenging due to its vulnerability and fixation difficulties because of its encapsulation by hard bone. Five cochleae were dissected out using diamond drills of various sizes in standardized surgical procedures. An experienced surgeon with the assistance of instrumental nurses was allowed to handle the specimens and delivered them to the fixative. Unless stored according to the Swedish biobank law, no data on the age, gender, or audiometry of the patients can be retrieved. After the cochleae were dissected from the surrounding bones, they were diluted in 4% paraformaldehyde with 0.1 M phosphate-buffered saline (PBS) (pH 7.4). The cochleae, transferred from the operating room to the laboratory, were kept in ample fixative fluid for 24 h at 4°C. Next, the specimens were washed in 0.1 M PBS and then placed in 10% Na-ethylene-diamine-tetra-acetic acid (Na-EDTA) solution at pH 7.2 for decalcification. The Na-EDTA solution was renewed every 2 days until the decalcification process was complete, which took ~3 weeks. The decalcified cochleae were rinsed with PBS and placed in 25% sucrose in PBS overnight (4°C). The cochleae were embedded in Tissue-Tek O.C.T. (Polysciences, Inc.), rapidly frozen in dry ice, and sectioned at 8–10 μm using a cryostat microtome (Leica, Tokyo, Japan). The cryo-sections were collected onto gelatin/chrome-alum-coated slides and stored in a freezer at −70°C before immunohistochemistry was conducted. The ESs were removed with a small rim of bone around the soft tissue. This tissue is normally drilled away during the routine trans-labyrinthine procedure to remove vestibular schwannomas.

### Antibodies and Immunohistochemistry

Table 1 shows the antibodies used in the present study. The immunohistochemistry procedures performed on the sections have been described in previous publications (19–21). Briefly, the slide-mounted sections were incubated with an antibody solution under a humidified atmosphere at 4°C for 20 h. After rinsing with PBS three times for 5 min each, the sections were incubated with secondary antibodies conjugated to Alexa Fluor 488, 555, and 647 (Molecular Probes, Carlsbad, CA, USA), counter-stained with the nuclear stain 4′,6-diamidino-2-phenylindole dihydro-chloride (DAPI; Thermo Fisher Scientific, Waltham, MA, USA) for 5–7 min, rinsed with PBS (3 × 5 min), mounted with ProLong® Gold Antifade Mountant (Thermo Fisher Scientific), and covered with the specified cover glass required for optically matching the SIM objectives. Primary and secondary antibody controls and labeling controls were performed to exclude endogenous fluorescence or unspecific reaction products. As a routine control, sections were incubated with 2% bovine serum albumin (BSA), omitting the primary antibodies. The control experiment revealed no visible staining in any structure of the cochleae.

**Table 1:** Antibodies used in this study.

**Primary antibody**	**Type**	**Dilution**	**Host**	**Catalog number**	**Producer**
IBA1	Polyclonal	1:100	Rabbit	PA5-27436	Thermo Fisher, Waltham, MA, USA
MHCII	Monoclonal	1:100	Mouse	MA5-11966	Thermo Fisher
Collagen IV	Polyclonal	1:10	Goat	AB769	Millipore, Burlington, VT, USA
CX3CL1	Monoclonal	1:50	Mouse	MAB3651-100	R&D Systems, Minneapolis, MN, USA
CD11b	Monoclonal	1:50	Rabbit	AB52478	Abcam, Cambridge, UK
CD4	Polyclonal	1:150	Goat	AF-379-NA	R&D Systems
CD8α	Monoclonal	1:100	Mouse	MAB1509	R&D Systems
CD68	Monoclonal	1:50	Mouse	NB100-683	Novus, Littleton, CO, USA
TLR 4	Oligoclonal	1:10	Rabbit	710185	Thermo Fisher
Tuj 1	Polyclonal	1:200	Rabbit	#04-1049	Millipore
Tuj 1	Monoclonal	1:200	Mouse	MAB1637	Millipore
TMEM119	Polyclonal	1:50	Rabbit	ab185337	Abcam

*Secondary antibodies used were the following*:

*Anti-mouse IgG (H+L), Alexa Fluor 555 Polyclonal 1:400 Goat A21422, Invitrogen*.

*Anti-rabbit IgG (H+L), Alexa Fluor 488 Polyclonal 1:400 Goat A11008, Invitrogen*.

*Anti-goat IgG (H+L), Alexa Fluor 488 Polyclonal 1:400 Donkey A21432, Invitrogen*.

*Anti-mouse IgG (H+L), Alexa Fluor 488 Polyclonal 1:400 Donkey A21202, Invitrogen*.

*Anti-rabbit IgG (H+L), Alexa Fluor 555 Polyclonal 1:400 Donkey A31572, Invitrogen*.

*Anti-goat IgG (H+L), Alexa Fluor 647 Polyclonal 1:400 Donkey A-21447, Thermo Fisher*.

### Imaging and Photography

To analyze sections, we used the methods earlier described by Liu et al. (14) The stained sections were first investigated with an inverted fluorescence microscope (Nikon TE2000; Nikon, Tokyo, Japan) equipped with a spot digital camera with three filters (for emission spectra maxima at 358, 461, and 555 nm). Image-processing software (NIS Element BR-3.2; Nikon, Tokyo, Japan), including image merging and a fluorescence intensity analyzer, was installed on a computer system connected to the microscope. For laser confocal microscopy, we used the same microscope equipped with a three-channel laser emission system. The optical scanning and image-processing tasks were performed using Nikon EZ-C1 ver. 3.80 software (Nikon, Tokyo, Japan) and included the reconstruction of Z-stack images into projections and three-dimensional (3D) images. SR-SIM, using an Elyra S.1 SIM system with a 63×/1.4 Oil Plan-Apochromat objective (Zeiss, Oberkochen, Germany), a sCMOS camera (PCO Edge), and ZEN 2012 software (Zeiss), was performed to investigate the structures of interest. Multichannel SR-SIM imaging was achieved with the following laser and filter setup: 405 nm laser of excitation coupled with BP 420–480 + LP 750 filter, 488 nm laser of excitation with BP 495–550 + LP750 filter, 561 nm laser of excitation with BP 570–620 + LP 750 filter, and 647 nm laser of excitation with LP 655 filter. To maximize image quality, five grid rotations and five phases were used for each image plane and channel. The grid size was automatically adjusted by the ZEN software for each wavelength of excitation. SR-SIM images were processed with the ZEN software with theoretical point spread function (PSF).

From the SR-SIM dataset, 3D reconstruction was performed with an Imaris 8.2 (Bitplane, Zürich, Switzerland). A bright-field channel was merged with fluorescence to visualize the cell borders. The microscope is capable of achieving a lateral (*X*–*Y*) resolution of ≈100 nm and an axial (*Z*) resolution of ≈300 nm (11). The resolution of the SIM system in BioVis (Uppsala University) was measured with sub-resolution fluorescent beads (40 nm) (Zeiss) in the green channel (BP 495–550 + LP750). An average PSF value was obtained from multiple beads with the built-in experimental PSF algorithm of the ZEN software. The typical resolution of the system was 107 nm in the *X*–*Y* plane and 394 nm in the *Z* plane. Next, 3D reconstructions of TUJ1 and IBA1 protein expression were conducted. Both signals were reconstructed by a surface rendering mode using Imaris 8.2 software. SIM is a wide-field technique that is based on the Moire effect of interfering fine striped patterns of excitation with sub-diffraction features in the sample emission. This can be compared with the confocal technique where the fluorescence light is detected only at the focal plane. This results in doubling the resolution and offers better possibilities to demonstrate proteins at a subcellular level. Combined with confocal microscopy, these techniques allow overviews of protein distribution in the tissue, as well as a more detailed cellular localization.

## Results

### SR-SIM of the Human ES (Figure 2)

**Figure 2:**
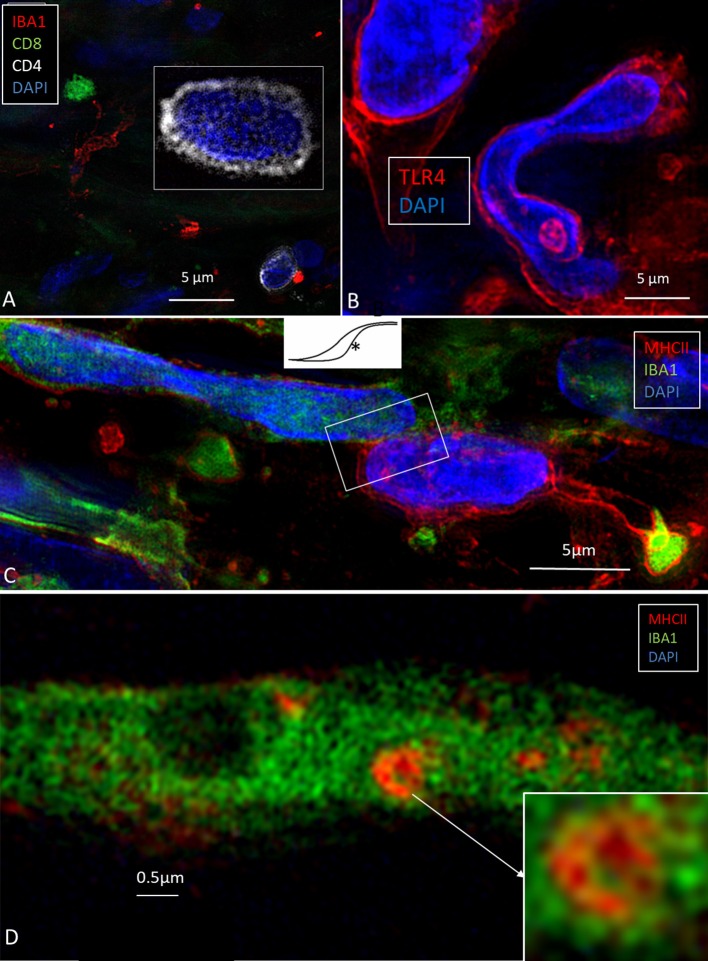
**(A)** SR-SIM of CD4- and CD8-positive cells present in the perisaccular tissue. **(B)** Some cells express the toll-like receptor 4 (TLR4). **(C)** Sub-epithelial cell interaction near the external aperture of the vestibular aqueduct. IBA1 cells interact (framed area) with cells strongly expressing MHCII. Cell nuclei show different protein expression [from Kampfe-Nordstrom et al. (12) with permission]. **(D)** A sub-epithelial IBA1 cell contains a multi-vesicular body expressing MHCII.

Ionized calcium-binding adaptor molecule 1-expressing cells (IBA1 cells) resided in the surrounding connective tissue and epithelium of the human ES. Macrophages interacted with other cells, showed migrant behavior, and expressed markers that suggest their active role in the innate and adaptive inner ear defense and tolerance (12). Macrophages, as well as some epithelial cells in the human ES, expressed major histocompatibility complex class type II (MHCII) mostly in the apical membrane. SR-SIM also revealed expression of toll-like receptor 4 (TLR4) in the cell membrane and in the cytoplasm among the sub-epithelial cells in the intermediate ES (Figure 2B). TLR4 was chosen since Møller et al. recently showed TLR4 and TLR7 expressed on the luminal side of the ES epithelium suggesting the ability to identify and trap bacterial antigens and virus RNA within the endolymphatic space (9). A few sub-epithelial cells expressed CD68, which was occasionally co-expressed with IBA1. The epithelium stained positive for the chemokine fractalkine. The expression was diffuse and intracellular, and occasionally, sub-epithelial fibrocytes also expressed fractalkine. Several migrating cells expressed CD68 and CD11b together with MHCII. Round cells expressing CD4 and CD8 were found in the ES, with more CD4+ than CD8+ cells (Figure 2A). Physical interaction between a CD4+ and an IBA1 cell was observed.

### IBA1 Cells in the Human Cochlea

**Figure 3:**
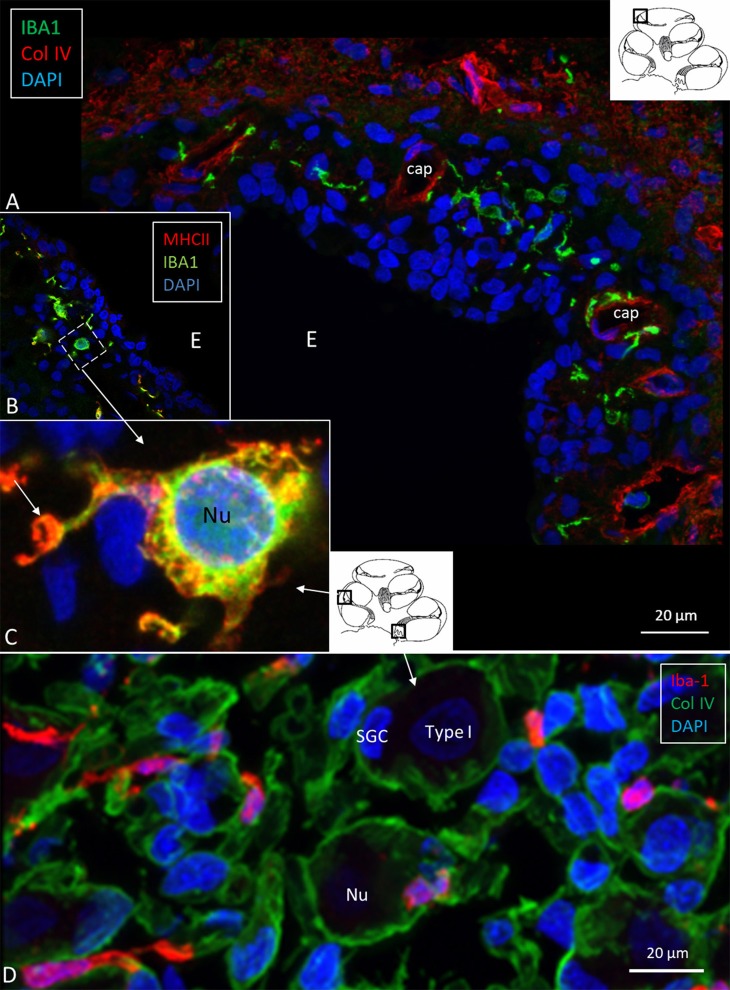
**(A)** Immunofluorescence of IBA1 and collagen IV in the lateral wall of the apical turn of the human cochlea. Many perivascular IBA1 cells are seen in the StV and few in the spiral ligament. **(B)** Confocal microscopy of the human StV. Framed area is magnified in **(C)**. **(C)** SR-SIM of framed area in B. Cell co-express IBA1 and MHCII. The cell membrane expresses MHCII as well as cytoplasmic vesicles [**(B,C)** from Kampfe-Nordstrom et al. (12)]. **(D)** Confocal microscopy of spiral ganglion with several surrounding IBA1 cells. SGC, satellite glial cell; Nu, type I cell nucleus; Col. IV, collagen IV; cap, capillary; E, endolymph.

SR-SIM demonstrated IBA1-positive cells in the lateral cochlear wall, including the spiral ligament, scala vestibuli (SV) and tympani (ST), spiral limbus, endosteum, tympanic covering layer (TCL), and spiral lamina. Even the organ of Corti (OC) occasionally contained active macrophages (14). In the lateral wall, most IBA1 cells were found in the epithelium of the stria vascularis (StV) near and around the blood vessels (Figure 3A). The cells expressed MHCII (Figures 3B,C, insets). IBA1 cells were present in the modiolus and cochlear nerve. A substantial number of mesenchymal cells surrounding spiral ganglion (SG) cells were in fact macrophages (Figure 3D) (14). The cells did not express TMEM119. Many IBA1-positive macrophages expressed MHCII in the StV and SG. The cells contained cytoplasmic aggregates of MHCII, and their slender processes often embraced the vessels. Fewer but similarly stained cells were detected in the spiral ligament. TLR4 was expressed in the StV (not shown).

### IBA1 Cells in the Human SG

**Figure 4:**
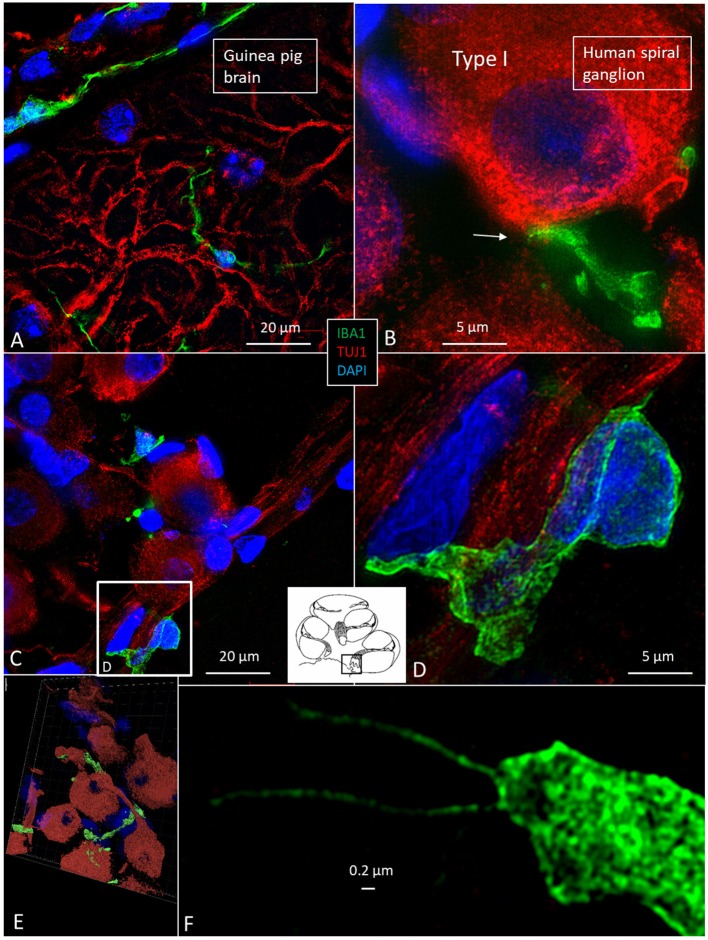
**(A)** SR-SIM of guinea pig brain showing IBA1-positive cells within the parenchyma and in a surrounding tissue sheet. **(B)** SR-SIM of human SGC and a surrounding contacting IBA1 cell (arrow). **(C,D)** show IBA1-positive cells, of which some are closely associated with the axon initial segment. **(E)** SGCs and IBA1 cells (shown in Video S1). **(F)** Nanoscopy of a peri-ganglionic IBA1 cell. Its surface coat contains “antenna”-like processes [from Liu et al. (14)].

The specificity of staining was compared to the guinea pig brain (Figure 4A). Several IBA1 cells were found in the human SG associated with the satellite cells (Figures 4B–E). IBA1 protein was expressed within the cytoplasm and in the cell nuclei (Figure 4D). The macrophages adhered to the basal lamina of the satellite cells located at the axonal and dendrite entry zones (Figures 4C–E; Video S1). At some places, the IBA1 cells seemed to perforate the basal lamina and reached the nerve cell membrane. “Synapse-like” endings faced the TUJ1-positive nerve soma (Figure 4B). Notable variants of IBA1 cells were found in Rosenthal's canal (RC). Free migrating cells were seen around and near the SGCs cells. They contained vesicles and thin (0.2 μm) remarkable processes projecting into the extracellular tissue (Figure 4F).

### Macrophages in Central and Peripheral Axons

**Figure 5:**
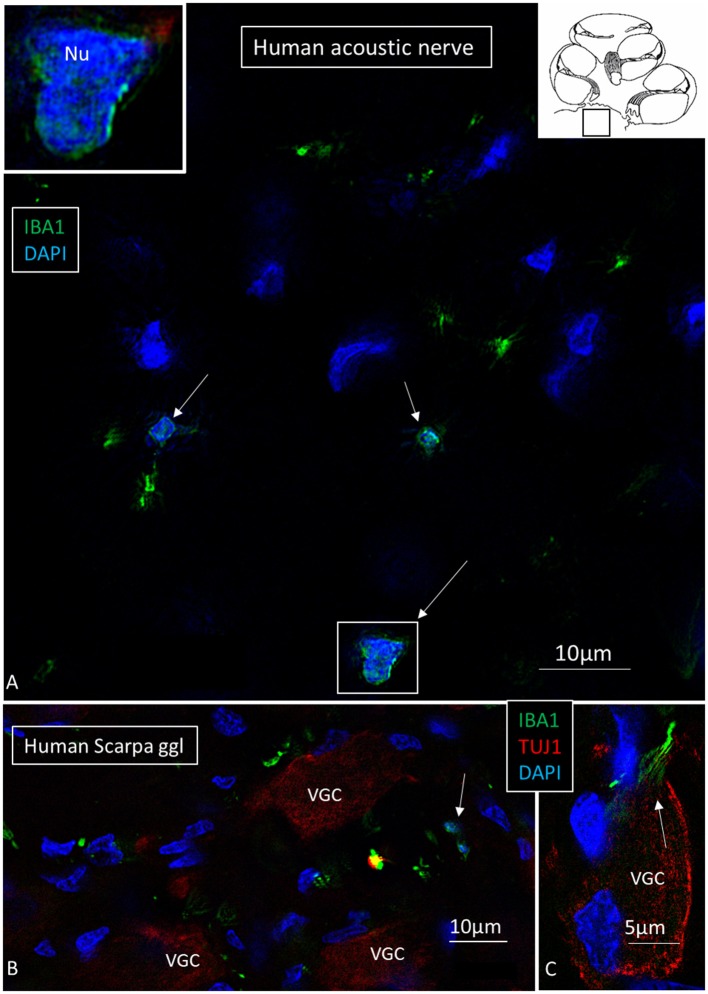
**(A)** SR-SIM of a cross-sectioned human cochlear nerve. Transected IBA1-positive cell processes are seen (arrows). Framed area is shown with higher magnification in inset. Its cell nucleus expresses IBA1. **(B,C)** Sectioned vestibular nerve at the level of the vestibular ganglion cells (VGCs) demonstrates several IBA1-positive cells (arrows). TUJ1: nerve marker tubulin-1.

**Figure 6:**
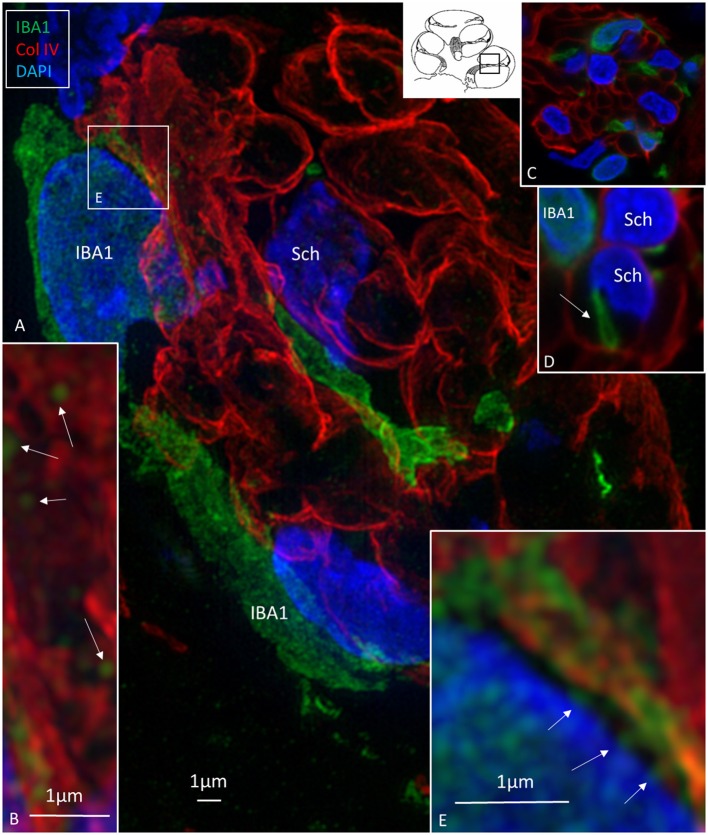
**(A)** SR-SIM (maximal intensity projection) of the osseous spiral lamina (framed area in inset). Collagen IV stains the basal lamina of the Schwann cells (Sch) surrounding the axons. Several IBA1-immunoreactive cells intermingle with the axons. **(B)** Thin processes (~150 nm in diameter) run along the Schwann cells. **(C,D)** The processes sometimes penetrate the basal lamina of the Schwann cells. **(E)** IBA1 protein expressed in the cell nucleus and at the nuclear envelope (arrows).

Macrophages were also physically related to axons and dendrites within RC and peripheral and central axons. IBA1 cells along the central axons were long and slender and measured up to 50 μm with a diameter of ~0.5 μm (Figure 5). Their nuclei expressed IBA1 (Figure 5A, left inset). The processes adhered to surrounding nerve fibers, and many had a terminal enlargement. Collagen IV and IBA1 co-staining showed that macrophage pseudopodia extended across the basal lamina of the Schwann cells in the osseous spiral lamina (Figure 6). The association with the myelin was uncertain. The IBA1 cells physically contacted Schwann cells' outer cell membrane (Figures 6C,D) (14). Whether or not the IBA1 branches directly adhered to the axonal cell membrane at the Ranvier nodes or intercellular clefts could not be determined with certainty. In several cells, IBA1 protein was associated with the nuclei pores (Figures 6A,E). At higher magnification, irregular stained areas (100–150 nm), representing cross-sectioned IBA1 branches, were noticed (Figure 6B). IBA1 cells ensued around the nerve fibers at the habenula perforata where nerves fibers lacked myelin. These cells did not enter the nerve perforation or reached the OC. Some cells extended along the TCL. The vestibular ganglion cells (VGCs) and axons were also surrounded by many IBA1-positive cells (Figures 5B,C).

### Expression of CX3C Chemokine Ligand 1 in the Cochlea

**Figure 7:**
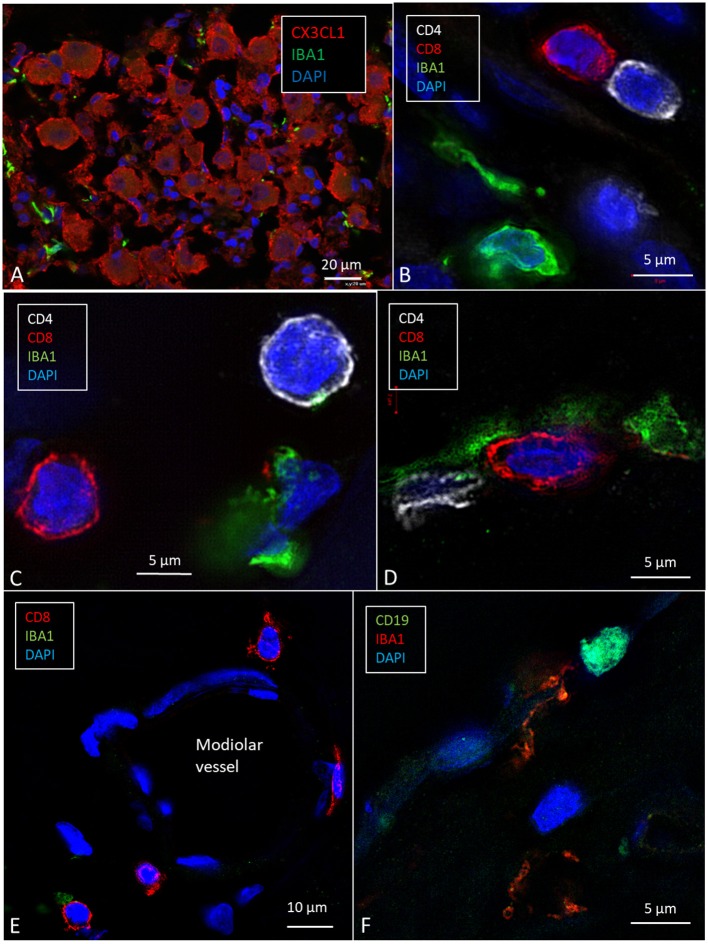
SR-SIM (maximal intensity projection) of the human spiral ganglion. **(A)** Several IBA1-positive cells surround the SGCs that express CX3CL1. **(B–D)** CD4- and CD8-positive lymphocytes are seen in Rosenthal's canal and around a modiolar blood vessel **(E)** [after permission from Liu and Rask-Andersen (16)]. **(F)** A cell in the modiolus expresses CD19.

Cells within the OC showed moderate expression of fractalkine. There was no difference in staining between hair cells and supporting cells. Cells of the TCL showed some staining, but the inferior surface of the basilar membrane lacked expression. SG cells strongly expressed fractalkine with some irregular membrane densities (Figure 7A).

### CD4+ and CD8+ Cells in the Human SG

A few CD4+ and CD8+ cells and their interactions with macrophages in the human cochleae were observed (Figures 7B–E). Some cells, together with occasional CD19-positive cell (Figure 7F), were located around modiolar blood vessels and along the border of RC (Figure 7E). The T cells were also seen in the medial wall between Rosenthal's canal and the ST. CD4+ and CD8+ cells were not found in the StV, or among the neurons in the Rosenthal's canal and the OC. A few isolated CD4+ and CD8+ cells were seen in the spiral ligament.

## Discussion

Our study confirms that the human inner ear and the eight cranial nerve contain a multitude of interacting IBA1-positive macrophages. O'Malley et al. (15) described cells expressing the macrophage markers CD163, IBA1, and CD68 in the connective tissue of the entire inner ear in normal human temporal bones. Some cells were even associated with neurons and the sensory epithelium. The location in the cochlear lateral wall suggests a function related to the “blood–labyrinth barrier” according to Zhang et al. (23) and Shi (24). Perivascular macrophages may control the exchange of agents across the vascular wall, but they have also been suggested to act as progenitors for postnatal vessels (24). In the brain and mouse spinal cord, these cells were shown to produce neurotrophic substances important for neuron survival (25, 26). Their highly variable morphology may reflect different functions and activation. We found no melanin in the cells, suggesting that they do not represent melanocytes or intermediate cells. According to Okano et al. (27), the cochlear macrophages appear to be monocyte-derived and do not represent microglia. We found no expression of TMEM119, a microglia marker in mouse and man (28), indicating that they were not microglia. The results support the findings by Hirose et al. (29, 30) and Sato et al. (31).

### Is There a Neuro-Immune Axis in the Human Cochlea?

**Figure 8:**
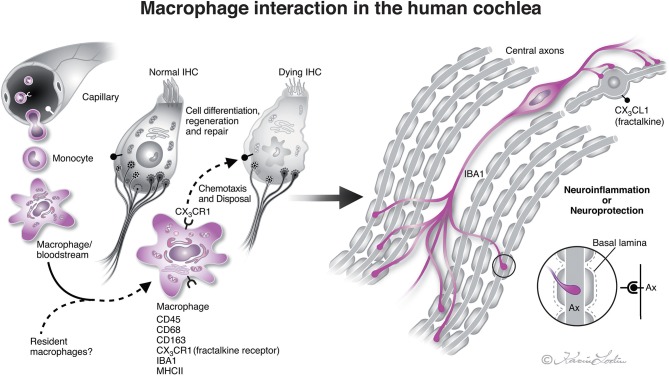
Illustration of nerve/macrophage interaction in the human cochlea [modified after (14)]. Macrophages that are positive for ionized calcium-binding adaptor molecule 1 (IBA1) cells are located in the human cochlea in the spiral ganglion and less often in the OC. They may interact and form a protective link between hair cells and neurons *via* a CX3CL1/CX3CR1 signaling, as demonstrated experimentally by Kaur et al. (22). Macrophages are believed to derive from blood-borne monocytes (illustration by Karin Lodin).

Many elongated IBA1 cells had terminal podosomes that attached to adjacent neurons in the modiolar auditory nerve. Torres-Platas et al. (32) analyzed human microglia in gray and white matter of the dorsal anterior cingulate cortex, a region associated with neuro-inflammation. They found a similar pattern of cells running along myelinated nerve fibers. Kaur et al. (22) and Hirose et al. (30) showed that inner ear lesions elevate the number of macrophages in the auditory nerve, spiral ligament, and spiral limbus. Chemokine signaling (fractalkine/CX3CL1) increased macrophage invasion and survival of auditory neurons after induced hair cell damage (22). A link may therefore exist between hair cells and neurons with a macrophage/neuron interaction that protects the cochlear nerve under various conditions (Figure 8). Also, adverse signaling may cause cochlear disease. In the human brain, microglial chemokine receptors may possibly promote adult neurogenesis by inhibiting Sirt 1/p65 signaling (33) or increasing secretion of neuroprotective BDNF (34). As a result, macrophages may act both as saviors and foes inducing damaging inflammatory reactions (M1-like) or immunosuppression (M2-like) (35), thus restoring tissue (35–37) and stimulating cell regeneration (38). Our results show that IBA1 cells may establish direct physical contacts with both vestibular and cochlear axons and ganglion cell bodies. Several studies of the human SG conducted in our laboratory over the years have suggested that these cells represent un-specified mesenchymal cells. The present results may help to explain human auditory nerve response following hair cell degeneration caused either by noise or ototoxic drugs (39) or as a result of aging. Macrophages may physically interact with the nerve cell body since they lack a surrounding compact layer of myelin. This may explain why, in contrast to most animals, the acoustic nerve is preserved after loss of hair cells and peripheral axons, a requisite for cochlear implantation (CI), which is one of the greatest achievements in modern medicine.

Notably, IBA1 cells in the human cochlea and auditory nerve expressed MHCII that was not found in experimental studies, unless tissues were induced by inflammation or γ-interferon (40, 41). Okano et al. (42) found bone-marrow-derived cells in the vestibular end organs and ES expressing MHCII. This suggests that CD4+ and CD8+ T cells may initiate adaptive immune responses from interaction with antigen-presenting cochlear macrophages. Conversely, a T-cell-induced inflammation may lead to hair cell damage and neuronal death, *via* pro-inflammatory cytokines and chemokines (25). Such responses need to be avoided. Both innate and adaptive neuro-inflammation with invasion of B- and T-lymphocytes may be responsible for the neurodegenerative process in Alzheimer's disease and MS (43). Moreover, microglia may attract peripheral immune cells and provoke adverse immune processes (44). Conversely, microglia may be neuroprotective through the production of neurotrophins (45, 46), and T cell autoimmunity has been found to even protect damaged neurons under certain conditions (47).

### The ES—An Immunologic Key Player?

A way to avoid mounting destructive inflammation around the sensory cells could be to let the ES monitor primary and secondary immune responses (3, 40). Altermatt found a few lymphoid cells expressing MHCII in the human ES epithelium collected post-mortem (48). The co-expression of IBA1 and MHCII in cells and their migratory behavior across the epithelium suggest that antigens may be taken up from the ES lumen (12) and processed. The apical cell membranes of the ES epithelial cells and cytoplasmic vesicles strongly expressed MHCII molecules. This is notable in the intestine where MHCII plays a role in mucosal immunology, modulation, and disease (49–51). Spectacular associations of MHCII molecular aggregates were seen in the ES among organelles, plasma membrane endocytosis, and multi-vesicular bodies (MVBs). Studies show that MVBs are involved in antigen proteolysis and peptide coupling to the MHCII complex (52). Antigen-presenting cells express MHCII on their surface and give proper information to CD4+ T helper cells and B cells (52, 53) to initiate adaptive immune responses. Gloddek et al. (41) showed the role of the peripheral circulation in response to inner ear antigen stimulation. Our finding of occasional lymphocytes in the cochlea raises the possibility of a “homing” of lymphocytes processed in the ES as suggested by Gloddek et al. (41). Antigens could reach the ES as a first defense line, followed by programmed memory cells entering the cochlea and auditory nerve. Thus, the inner ear could be protected without initiating a full-scales and harmful immune cascade around the receptors. In earlier studies, lymphocytes were observed in the ST and around the spiral modiolar vein after immune challenges to the cochlea (41). This suggests that the vein is the initial site for lymphocytes entering the inner ear (54).

### Cochlear Macrophages and Cell Renewal

In a recent study, we found migratory macrophages in the human cochlea near injured hair cells (14). These scavenger cells were thought to stimulate repair *via* supporting cells. Furthermore, active macrophages could be observed within the sensory epithelium after noise damage, suggesting that they are involved in tissue reconstruction (29, 55, 56). In the eye, macrophages, microglia, and T cells have been shown to enhance the survival of retinal ganglion cells and even regenerate damaged axons through the inflammatory response (57). Moreover, bone-marrow-derived cells, chiefly hematopoietic stem cells, were found to continuously populate the lateral wall in the adult cochlea (58). The authors believed that these cells can regenerate damaged fibrocytes and differentiate into macrophages in the adult auditory nerve. They even suggested that the cells may constitute a source for regeneration of the human acoustic nerve in the adult inner ear (59).

Stem-cell-based regeneration of sensorineural elements in the ear may be hindered by immune responses. The blood–labyrinth barrier may restrict cell migration and consists partly of endothelial tight junctions in the StV. The SG and ES contain fenestrated capillaries and lack a corresponding constricted barrier. In the central nervous system, monocyte-derived IBA1 cells expressing MHCII seem to respond to mesenchymal stem-cell grafting, even though resident microglia may also be involved (60). If similar restrictions prevail after inner ear nerve grafting remains to be elucidated.

In summary, our freshly fixed human specimens showed unique preservation and immunogenicity. The benign tumors could potentially influence the conditions. Tumor infiltration into the cochlea was not noticed, and we believe that the samples are physiologically representative. The results also affirm the findings by O'Malley et al. (15). However, a weakness of the study may be the age of the patients (~40–60 years), as microglia of the aged brain can show an increased immune state (61).

## Data Availability

All datasets generated for this study are included in the manuscript and/or the Supplementary Files.

## Author Contributions

WL and CK performed all the immunohistochemistry and processing of the human tissue, such as fixation, embedding, and cryo-sectioning. They also did confocal and SIM microscopy together with HR-A. HR-A is the main writer of the manuscript, and he also edited the figures, designed and supervised the research project, and participated in the research procedures and interpretation of the results and the photography. ND-L performed the surgery.

### Conflict of Interest Statement

Medel Inc., Innsbruck, Austria, generously contributed a portion of WL's salary. The remaining authors declare that the research was conducted in the absence of any commercial or financial relationships that could be construed as a potential conflict of interest.

## Supplementary Material

Video S1 3D reconstruction showing IBA-positive cells among TUJ1-positive neurons (red) in the human SG. Signals were remodeled using Imaris 8.2 software.

## Abbreviations

CI: cochlear implant
E: endolymph
EDTA: ethylene-diamine-tetra-acetic acid
IBA1: ionized calcium-binding adaptor molecule 1
MHCII: major histocompatibility complex type II
SR-SIM: super-resolution structured illumination fluorescence microscopy
ST: scala tympani
StV: stria vascularis
SV: scala vestibuli
TEM: transmission electron microscopy
ES: endolymphatic sac
PBS: phosphate-buffered saline
BSA: bovine serum albumin
TLRA: toll-like receptor
TCL: tympanic covering layer
OC: organ of Corti
VGCs: vestibular ganglion cells
CI: cochlear implant
MVBs: multi-vesicular bodies
FN: facial nerve
IAM: internal acoustic meatus
Na-EDTA: sodium-ethylene-diamine-tetra-acetic acid
DAPI: 4′,6-diamidino-2-phenylindole dihydro-chloride
SG: spiral ganglion
SGCs: spiral ganglion cells
RC: Rosenthal's canal
CX3CL1: CX3C chemokine ligand 1
Sch: Schwann cells
Col. IV: collagen IV.
